# *In Utero* Exposure to Exosomal and B-Cell Alloantigens Lessens Alloreactivity of Recipients’ Lymphocytes Rather than Confers Allograft Tolerance

**DOI:** 10.3389/fimmu.2018.00418

**Published:** 2018-03-02

**Authors:** Jeng-Chang Chen, Liang-Shiou Ou, Cheng-Chi Chan, Ming-Ling Kuo, Li-Yun Tseng, Hsueh-Ling Chang

**Affiliations:** ^1^Department of Surgery, Chang Gung Children’s Hospital, College of Medicine, Chang Gung University, Taoyuan, Taiwan; ^2^Division of Allergy, Asthma and Rheumatology, Department of Pediatrics, Chang Gung Children’s Hospital, College of Medicine, Chang Gung University, Taoyuan, Taiwan; ^3^Department of Microbiology and Immunology, College of Medicine, Graduate Institute of Biomedical Sciences, Chang Gung University, Taoyuan, Taiwan; ^4^Pediatric Research Center, Chang Gung Children’s Hospital, Taoyuan, Taiwan

**Keywords:** alloreactivity, B-cells, exosome, *in utero* injection, major histocompatibility complex, tolerance induction

## Abstract

According to actively acquired tolerance, antigen exposure before full immune development in fetal or early neonatal life will cause tolerance to this specific antigen. In this study, we aimed to examine whether allogeneic tolerance could be elicited by *in utero* exposure to surface MHC antigens of allogenic cells or soluble form of MHC exosomes. Gestational day 14 FVB/N fetuses were subjected to intraperitoneal injection of allogeneic major histocompatibility complex (MHC) exosomes or highly enriched B-cells. Postnatally, the recipients were examined for the immune responses to donor alloantigens by lymphocyte proliferative reactions and skin transplantation. *In utero* exposure to allogeneic MHC exosomes abolished the alloreactivity of recipients’ lymphocytes to the alloantigens, but could not confer skin allograft tolerance. *In utero* transplantation of highly enriched allogeneic B-cells generated low-level B-cell chimerism in the recipients. However, it only extended the survivals of skin allograft by a few days despite the lack of donor-specific alloreactivity of recipients’ lymphocyte. Thus, an early *in utero* contact with exosomal or B-cell alloantigens did not lead to full skin tolerance but rather, at best, only to delayed skin rejection in the presence of microchimerism made by B-cell inocula. These results argued against the theory of actively acquired tolerance, and implicated that *in utero* exposure to marrow cells in previous studies was a unique model of allo-tolerance induction that involved the establishment of significant hematopoietic chimerism. Taken together with the discovery of *in utero* sensitization to ovalbumin in our previous studies, the immunological consequences of fetal exposure to foreign antigens might vary according to the type or nature of antigens introduced.

## Introduction

According to Medawar’s actively acquired tolerance (1), the immune system before full maturation undergoes a critical education so as to learn the discrimination between self and non-self. Based on this knowledge, antigen exposure during the critical education period of fetal or early neonatal life will cause tolerance to this specific antigen. Thus, the prenatal life may represent a favorable period for the implementation of medical interventions that will be later hampered by immune responses. Such an idea has attracted widespread attention of transplantation community to prenatal allo-tolerance induction across major histocompatibility complex (MHC) barriers. The key targets of transplantation immune reactions are the cell surface MHC antigens, of which a matching between donors and recipients significantly improves graft acceptance (2, 3). As a consequence, MHC molecules or their related constituents may be used as biological reagents to endow fetal recipients with allo-tolerance. During the 1960s, nodal or splenic lymphocytes were considered as an excellent tolerogenic reagent to render the immunologically immature fetus or neonate tolerant of skin allografts (4, 5). However, these early studies had used the murine strain combinations with minimal or even absent MHC disparity. The weak host-versus-graft reactions could not reflect the reality in clinical arena with almost fully MHC-mismatched transplants. More importantly, the claimed superiority of nodal or splenic lymphocytes apparently overlooked the detrimental effects of allogeneic T-cells that might cause postnatal graft-versus-host disease following *in utero* transplantation even without the employment of myeloablation or immunosuppression (6–10). Notably, immunologically incompetent fetuses were even more vulnerable to the attack from allogeneic T-cells than anticipated, as evidenced by the observation that fully MHC-mismatched lymphocytes rapidly elicited lethal graft-versus-host disease in fetal recipients (11). As a result, it is risky to use cell inocula containing allogeneic T-cells for prenatal allo-tolerance induction. Thus, an ideal source of alloantigens for prenatal tolerance induction whenever possible will be the cell inocula without T-cells or surface MHC molecules related to transplantation rejection. Soluble forms of MHC have been described in mouse and human sera (12, 13) as cell-derived secretory vesicles of exosomes (14, 15), derived from antigen-presenting cells (APCs), such as dendritic cells (16–19), B-cells (20), and mast cells (21, 22). Their transfer to hosts through transfusion has been suggested to result in immunomodulatory effects (23). Thus, it prompted us to examine whether B-cell inocula or soluble form of MHC exosomes were effective in prenatal induction of donor-specific tolerance.

## Materials and Methods

### Ethics Statement

This animal study was conducted in accordance with the standards, guidelines, and regulations as laid down in “Guide for the Care and Use of Laboratory Animals,” Chang Gung Memorial Hospital (CGMH). All protocols were approved by the CGMH Committee on Animal Research.

### Cell Lines Culture

The A20 cell line is a BALB/C B-cell lymphoma line derived from a spontaneous reticulum cell neoplasm found in an old BALB/C AnN mouse (24). The cells can present both alloantigens and protein antigens (25). For generation of supernatants rich in exosomes, this murine A20 B-cell line was maintained by growth in RPMI 1640 plus 10% exosome-depleted fetal calf serum for 3 days at a concentration of 5 × 10^5^ cells/ml. The culture supernatant was collected for the enrichment of exosomes.

### Ultracentrifugation and Exosome Isolation (20)

To fractionate exosomal antigens from cell line culture, supernatants were first spun at 300 x *g* for 10 min, 4 °C to deplete cells and then at 2,000 x *g* for 10 min, 4 °C to deplete residual cellular debris. Samples were then transferred to polyallomer tubes for ultracentrifugation at 10,000 x *g* for 30 min, 4 °C. The supernatant was transferred to a fresh tube of the same size as the previous step for further enriching exosome fraction with ultracentrifugation at 100,000 x *g* for 70 min, 4 °C. Then, the supernatant was poured off completely to obtain the exosome fraction. The pellet from each tube was resuspended in 1 ml PBS using a micropipettor. All the resuspended pellets were pooled in a single centrifuge tube. The pooled exosome fraction was further spun at 100,000 x *g* for 70 min, 4 °C. The pellet was then collected and resuspended at a small volume of 100–200 µl normal saline. BALB/C MHC exosome was then examined under transmission electron microscopy after processing, quantified by BCA protein assay, and adjusted at a concentration of 100–300 µg/ml.

### MHC Exosome Identification by Western Blotting

Major histocompatibility complex exosome concentrate was 0.22-µm filtered before immunoblotting. The exosome sample of 10 µl was mixed with 10 µl Laemmli sample buffer (Bio-Rad), separated by 10% SDS-polyacrylamide gel electrophoresis (SDS-PAGE), and transferred to an Immobilon-P membrane (Millipore) in buffer containing 25 mM Tris, 192 mM glycine, 1% SDS, and 20% methanol. After blocked with 5% BSA in 0.1% Tween-20/PBS for 1 h, the membranes were probed with mouse anti-mouse H-2K^d^ antibody (Clone SF1-1.1, 1:1,000, BioLegend) at room temperature for 2 h, washed three times with 0.1% Tween-20/PBS, and then incubated with HRP-conjugated goat anti-mouse IgG (H+L) (1:10,000, Millipore) at room temperature for 2 h. Finally, immunoblots were washed three times, developed with Chemiluminescent HRP substrate (Thermo Scientific Pierce ECL Western Blotting Substrate) for 5 min, and then exposed to films for 1 min.

### Quantification of MHC Exosome by Bicinchoninic Acid (BCA) Protein Assay (Thermo Scientific)

Bicinchoninic acid working reagent was prepared by mixing 50 parts of BCA Reagent A with 1 part of BCA Reagent B (50:1, Reagent A:B). 25 µl of each standard or unknown sample replicates were pipetted into a microplate well. Then, 200 µl of working reagent were added to each well and mixed thoroughly on a plate shaker for 30 s. The plate was covered and incubated at 37°C for 30 min. Optical density readings at 562 nm on a plate reader were quantified into unit values against a standard curve prepared from diluted albumin standards (0, 25, 125, 250, 500, 750, 1,000, 1,500, and 2,000 µg/ml).

### Mouse Husbandry

Inbred FVB/N mice were purchased from National Laboratory Animal Center (Taipei, Taiwan) at the age of 6–8 weeks, and housed in the Animal Care Facility at CGMH with the approval of the CGMH Committee on Animal Research. Females were caged with males in the afternoon and checked for vaginal plugs the following morning. The day of the plug observed was called day 0 of the pregnancy.

### Preparation of B-Cells

Spleens from C57BL/6 mice were dissociated by passage through a 70-µm cell strainer. Splenic lymphocytes were then obtained by layering them over NycoPrep 1.077A and centrifuging at 600 × *g* for 25 min, and then washed with PBS. B-cells were enriched by negative selection using mouse Pan B Cell Isolation Kit (Miltenyi Biotec) according to the manufacturer’s instruction. The purity of enriched B-cells was examined by flow cytometry using fluorescence-conjugated antibodies to CD3 and CD19/CD45R (Biolegend).

### *In Utero* Injection of BALB/C MHC Exosomes or C57BL/6 B-Cells

Under anesthesia with ketamine (100 mg/kg) and xylazine (10 mg/kg), the uteri of gestational day 14 pregnant mice (FVB/N) were exposed through a vertical laparotomy. A 60-µm glass micropipette with beveled tip was used to inject the transplant inoculum, BALB/C exosome extract (1–3 µg) or enriched C57BL/6 B-cells (2.5–5.0 or 5.0–7.5 × 10^6^) in 10 µl. The tip of the micropipette was inserted through the uterine wall into the peritoneal cavity of each fetus of the same litter to deliver the inocula. Then the abdomen of the pregnant mice was closed using two layers of 5-0 silk suture. After the operation, all mice were housed in an undisturbed room without bedding changes until the pups were 1 week old. Pups were weaned at 3 weeks of age. The control mice received normal saline injection.

### Analyses of Chimerism and Lineages

Peripheral blood was sampled from tail veins. The examination of chimerism in other tissues or peritoneum demanded the sacrifices of recipients. Peritoneal cells were harvested by flushing the peritoneal cavity with 10 ml PBS. Bone marrow cells (BMCs) were harvested by flushing the tibias and femurs with PBS using a 26-G needle. Thymuses and spleens were obtained, washed with PBS, and dissociated by passage through a 70-µm cell strainer. Red cells were removed by ACK lysing buffer. Donor chimerism was assessed, using anti-CD45R FITC and anti-H-2K^b^ PE antibodies (Biolegend) after blockage of Fc receptors by anti-mouse CD16/32 antibody (Clone 93, Biolegend). Cells were acquired for analysis after gating out dead cells using propidium iodide.

### Proliferative Response of Lymphocytes

Splenic lymphocytes from FVB/N recipients or their controls were enriched by density gradient centrifugation, and then cultured in triplicate each with 2 × 10^5^ cells in 200 µl RPMI 1640 medium containing 10% fetal calf serum. Responder lymphocytes were stimulated with BALB/C exosome (0.025–20 µg/ml), Con-A (2 µg/ml) or 3,000 cGy irradiated lymphocytes (6 × 10^5^ cells) from FVB/N, C57BL/6, or BALB/C mice. Lymphocyte proliferation was measured in day 5 cultures. Tritiated thymidine (ICN Biomedicals) was added at a final concentration of 1 μCi per well. Next day, cells were harvested for counting incorporated tritium in a liquid scintillation counter (1450 Microbeta Plus counter).

### Skin Transplantation

Tolerance was examined by skin transplantation at the age of 4–8 weeks, using tail skins from BALB/C (exosome donor strain) or C57BL/6 (B-cell donor strain) mice. After dressing removal on post-transplant day 7, skin grafts were monitored daily until they were rejected or engrafted for at least 4 months. Engrafted skin was defined by good hair growth. Rejection was defined as when less than 20% of the original graft remained. A tolerant state was defined by skin engraftment for at least 120 days.

### Statistical Analyses

The data of all groups were first subjected to the evaluation of normal distribution by Shapiro–Wilk or Kolmogorov–Smirnov tests. If the tested variables followed a normal distribution, the equality of means was examined by Student’s *t-*test between two independent groups, or one-way ANOVA among three or more groups with a *post hoc* multiple comparison test by least significant difference; otherwise, the significance of differences was analyzed by non-parametric tests. As for survival analyses of skin transplants, the graft survival time was defined by estimating the length of time from the date of skin transplantation to the date of graft rejection. Plots of survival time were constructed by Kaplan–Meier method. The log rank test was employed to compare survival curves. Differences were regarded as significant in all tests at *P* < 0.05.

## Results

### Exosome Generation

**Figure 1:**
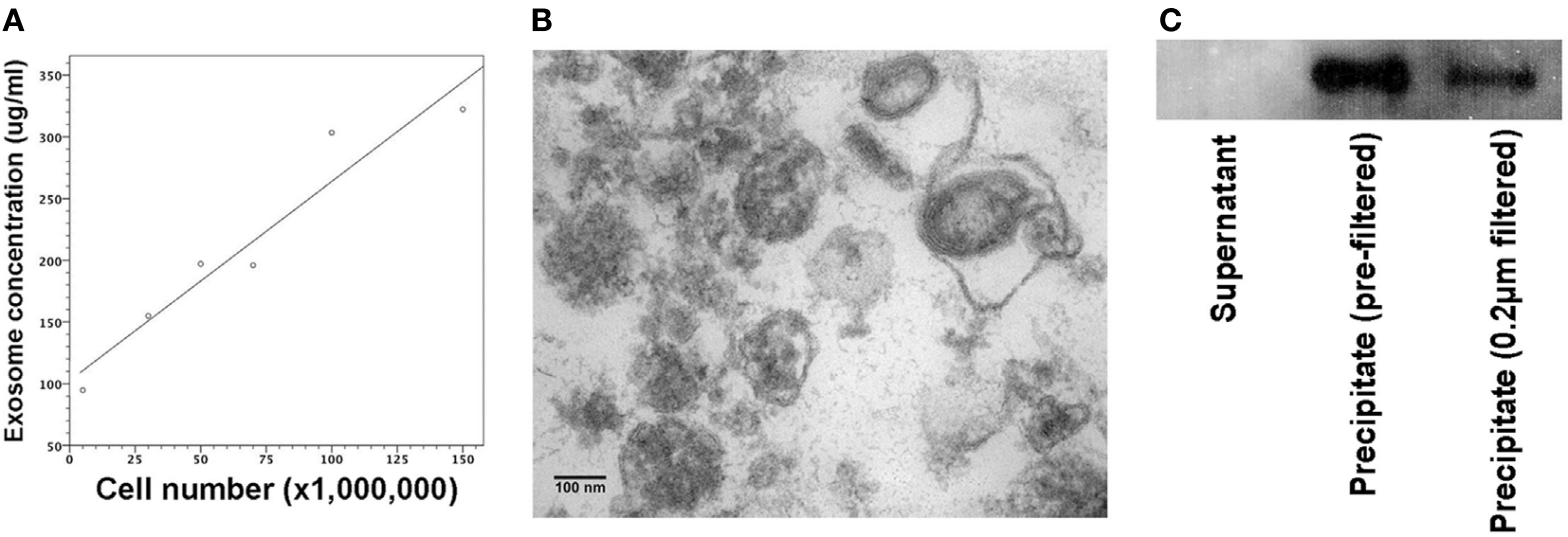
Quantification and verification of major histocompatibility complex (MHC) exosomes. Exosomes were generated from A20 cell line at the concentration of 5 × 10^5^ A20 cells/ml. Following the centrifugation and ultracentrifugation, the final supernatant and pellet were collected, respectively. **(A)** BCA assay showed that protein concentration of resuspended pellets was proportional to the A20 cell number used for exosome generation. Pearson’s correlation coefficient (0.961) was significant at the 0.01 level with *P*-value of 0.002. **(B)** Under the transmission electron microscope, there were bilayer membrane-bound vesicles, sized around 100 nm in the resuspended pellet. **(C)** Immunoblotting demonstrated the expression of MHC class I (H-2K^d^) in the resuspended pellet regardless of 0.22-µm filtration. Controls were samples from the supernatant after the final ultracentrifugation.

A20 cells were grown in RPMI media at a concentration of 5 × 10^5^/ml. We collected supernatants of cell cultures containing 5 × 10^6^, 3 × 10^7^, 5 × 10^7^, 7 × 10^7^, 1 × 10^8^, and 1.5 × 10^8^ A20 cells. Following centrifugation and ultracentrifugation, exosome extracts were quantified by BCA protein assays with its protein concentration linearly associated with A20 cell number used for exosome generation (Figure 1A). MHC exosomes were further verified by the electron microscope and immunoblotting, showing the morphology of about 100 nm bilayer membrane-bound microvesicles (Figure 1B) and the expression of MHC class I H-2K^d^ (Figure 1C).

### Proliferation Responses of FVB/N Lymphocytes to BALB/C Exosome

**Figure 2:**
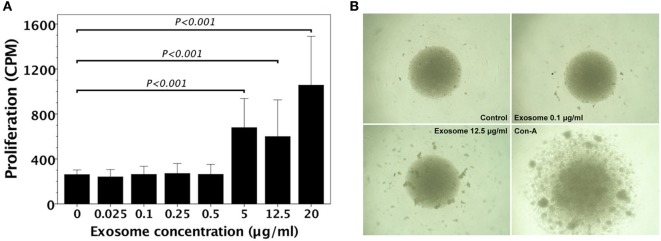
FVB/N lymphocyte proliferation in response to H-2^d^ (A20 cell line) major histocompatibility complex (MHC) exosomes. **(A)** FVB/N lymphocytes were cultured with various doses of H-2^d^ MHC exosomes for 5 days. The controls were lymphocyte cultures without adding H-2^d^ MHC exosomes. The proliferative response (*n* = 3) was measured by the readout of incorporated tritium as counts per minute (CPM). The proliferative response was evident when there was the exosome concentration of ≥5 μg/ml. **(B)** Proliferative colonies of FVB/N lymphocytes in response to H-2^d^ MHC exosomes were observed under an Olympus BX50 microscope.

We examined whether FVB/N (H-2^q^) lymphocytes had allogeneic proliferative responses to MHC exosomes. A variety of exosome concentrations were used to stimulate allogeneic proliferation of FVB/N lymphocytes. Proliferation of FVB/N lymphocytes to H-2^d^ MHC exosomes did not show up until 5 µg/ml exosome extract was added (Figure 2A). Proliferative colonies could be visualized under a microscope (Figure 2B).

### Immune Responsiveness of FVB/N Mice after *In Utero* Injection H-2^d^ Exosome

**Figure 3:**
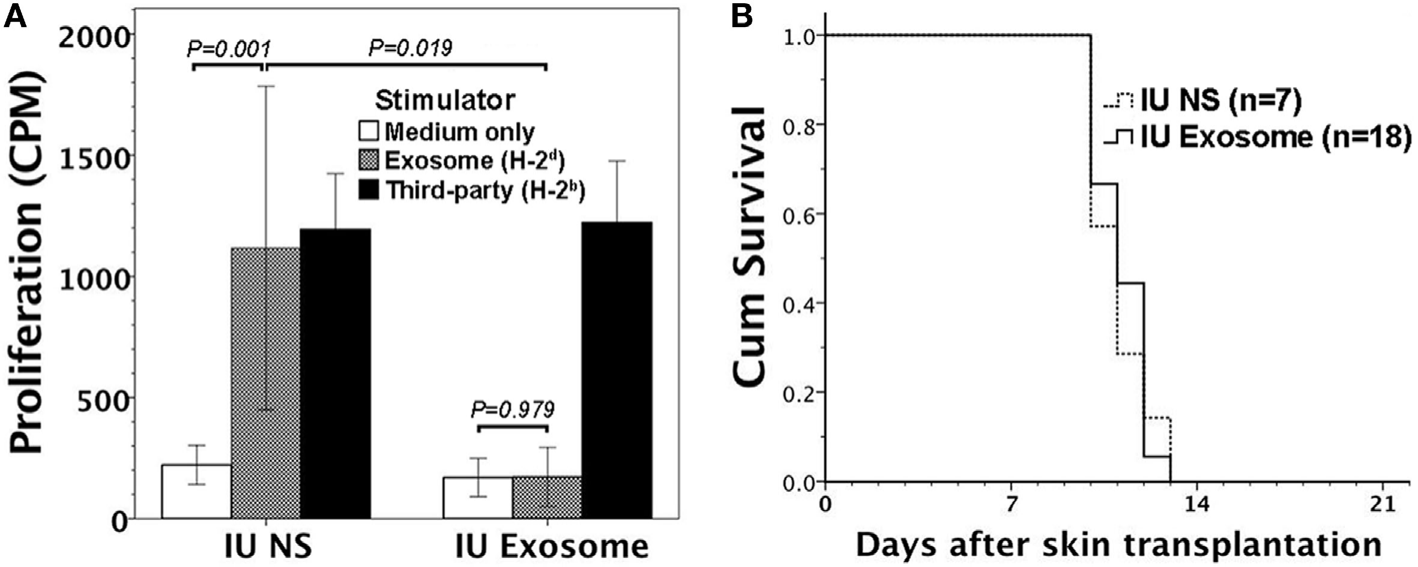
Immune reactivity to H-2^d^ alloantigens in FVB/N mice with *in utero* exposure to H-2^d^ exosomes. Gestational day 14 FVB/N murine fetuses were subjected to *in utero* exposure to H-2^d^ exosomes from A20 cell lines (IU exosome). **(A)** Postnatally (at 6–8 weeks old without skin transplantation), their lymphocytes did not exhibit proliferative responses *in vitro* to H-2^d^ exosomes (*P* = 0.979, *n* = 3), whereas the lymphocytes of the controls with *in utero* saline injection (IU NS) significantly proliferated (*P* = *0.001, n* = 3). Lymphocyte proliferation in response to exosomes also reached statistical significance between IU exosome and IU NS (*P* = 0.019). **(B)** FVB/N recipient mice were also subjected to transplantation of BALB/C (H-2^d^) allogeneic skin. All the allogeneic skin grafts (*n* = 18) were rejected within 14 days after transplantation, similar to the graft survivals of IU NS (*P* = 0.800, *n* = 7).

Gestational day 14 FVB/N murine fetuses were subjected to intraperitoneal injection of 1–3 μg H-2^d^ exosome extract. Postnatally, recipient mice (6–8 weeks old) were evaluated for their immune responsiveness to H-2^d^ MHC alloantigens by *in vitro* lymphocyte culture, and *in vivo* skin transplantation. In mice with prenatal exposure to H-2^d^ exosomes, their lymphocytes did not proliferate in response to H-2^d^ exosome extract as compared with saline control mice (Figure 3A). However, the FVB/N recipients could not be rendered tolerant to BALB/C (H-2^d^) skin grafts (Figure 3B) after skin transplantation.

### Donor Cell Chimerism after *In Utero* Injection of C57BL/6 B-Cells into FVB/N Murine Fetuses

**Figure 4:**
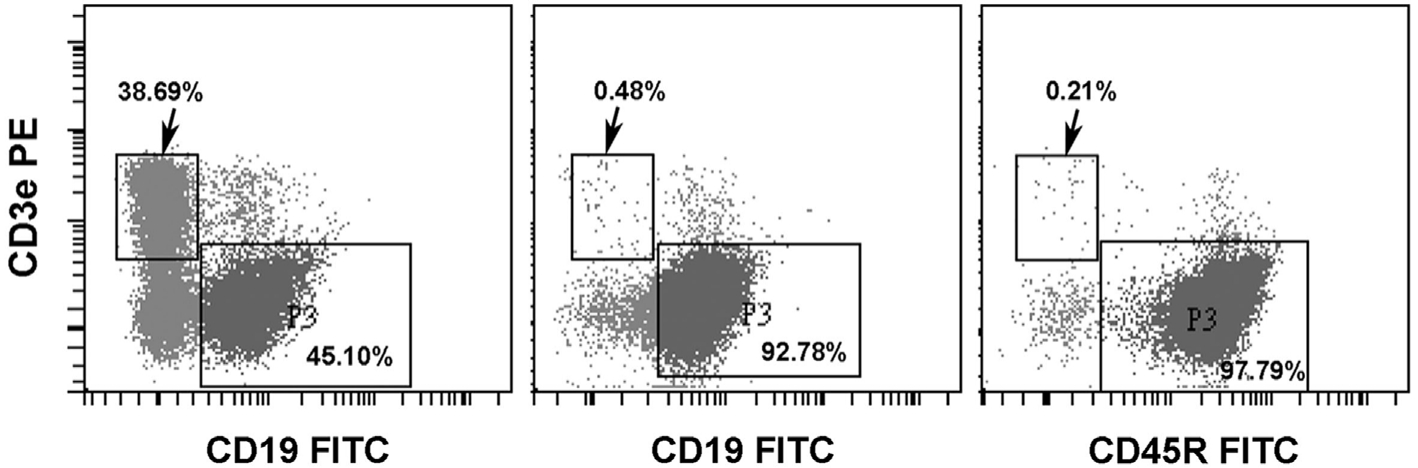
Enrichment of splenic B-cells. C57BL/6 splenic B-cells were negatively selected by Pan B Cell Isolation Kit (Miltenyi Biotec). A representative experiment showed that CD3 T-cells and CD19 B-cells were 38.69 and 45.10%, respectively, in splenic lymphocytes before enrichment (left panel). Following negative selection, CD3 T-cells were less than 0.5% and B-cells examined by either anti-CD19 (middle panel) or anti-CD45R (B220) (right panel) were above 90%.

In our previous studies, donor T-cells were found to be extremely detrimental to the pre-immune fetuses because of their fatal graft-versus-host effects (11). In order to evaluate the tolerogenic effects on pre-immune fetuses by cell surface alloantigens without the influence of graft-versus-host effects, we then employed allogeneic B-cells as the source of cell surface alloantigens for *in utero* injection. B-cells were negatively selected from C57CL/6 splenocytes. Among the enriched B-cells, CD3 T-cells were less than 0.5% and either CD19 or CD45R B-cells were over 90% (Figure 4).

**Figure 5:**
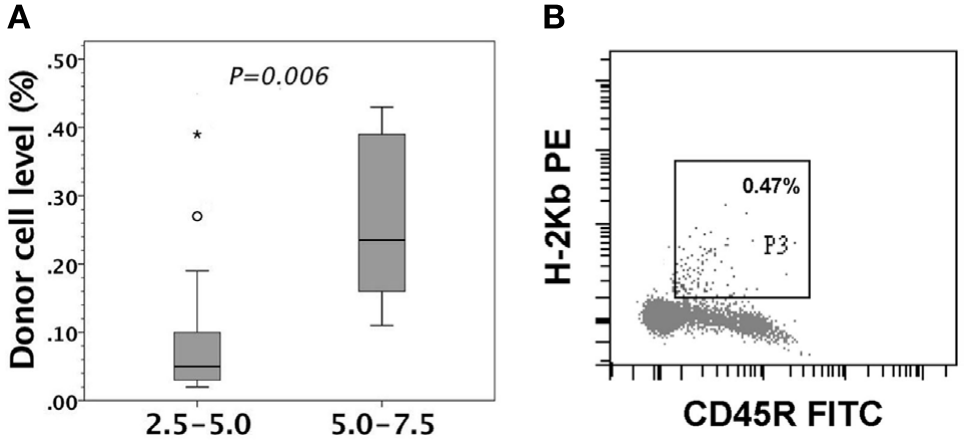
Peripheral chimerism after *in utero* injection of C57BL/6 (H-2^b^) B-cells into FVB/N (H-2^q^) fetuses. FVB/N recipients were examined for peripheral chimerism at their age of 4–6 weeks. **(A)** 5.0–7.5 × 10^6^ B-cells (5.0–7.5, *n* = 6) generated significantly higher peripheral chimerism (*P* = *0.006*) than 2.5–5.0 × 10^6^ B-cells (2.5–5.0, *n* = 14). The significance of differences was measured by non-parametric Mann–Whitney *U* test. The boxplot shows the median as a horizontal line inside the box and the interquartile range (between the 25 and 75th percentiles) as the length of the box. The whiskers (line extending from the top and bottom of the box) represent the minimum and maximum values when they are within 1.5 times the interquartile range from either end of the box. A score greater than 1.5 times the interquartile range is out of the boxplot and is considered as outliers (circle), and that greater than three times the interquartile range is extreme outliers (asterisk). **(B)** A representative recipient had the low-level chimerism of 0.47% (H-2K^b+^) in the circulation. The engrafted donor cells were shown to be CD45R-positive, indicating donor B-cell origin.

Gestational day 14 FVB/N murine fetuses received *in utero* injection of 2.5–5.0 or 5.0–7.5 × 10^6^ enriched C57BL/6 B-cells. We collected 14 and 6 recipient mice, respectively, in the two groups to examine their peripheral chimerism at the age of 4–6 weeks by flow cytometry. B-cells of 5.0–7.5 × 10^6^ generated significantly higher peripheral chimerism than those of 2.5–5.0 × 10^6^ (Figure 5A). Engrafted donor cells in the peripheral blood were CD45R B-cells (Figure 5B). We also subjected 11 recipients to the examinations of donor cell chimerism in peritoneum, bone marrow, thymus, and spleen. There was no predilection site to harbor a considerable number of donor B-cells. Their levels ranged from undetectable to less than 0.5% (data not shown).

### Skin Transplantation after *In Utero* Injection of C57BL/6 B-Cells

**Figure 6:**
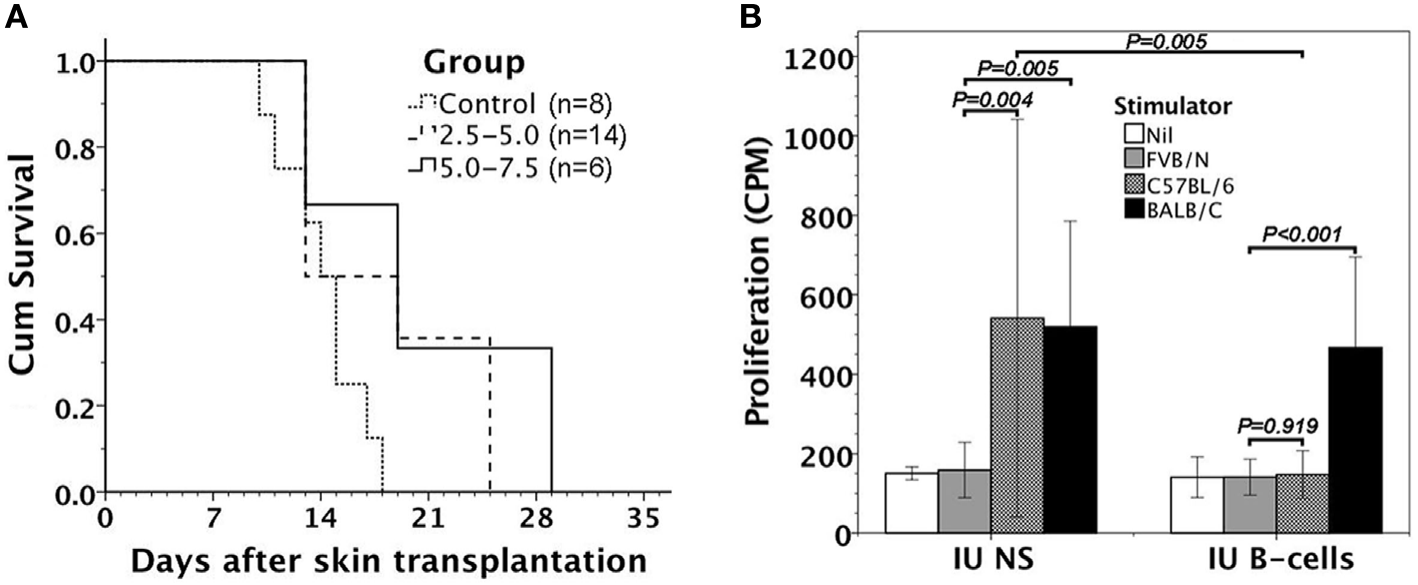
Alloreactivity of FVB/N mice after *in utero* injection of C57BL/6 B-cells. **(A)** Following donor skin transplantation, FVB/N recipients had better survivals of C57BL/6 skin grafts than the controls with *in utero* saline injection (*P* = 0.029 for 2.5–5.0 and *P* = 0.025 for 5.0–7.5). However, it made no difference in graft survivals between two B-cell doses of 2.5–5.0 × 10^6^ and 5.0–7.5 × 10^6^ used (*P* = 0.273). **(B)** Mixed lymphocyte reactions in response to FVB/N, C57BL/6, and BALB/C antigens were examined. Lymphocytes from FVB/N recipients with *in utero* B-cell injection (IU B-cells, *n* = 5) were responsive to third-party BALB/C stimulators (*P* < 0.001), but not to donor-specific C57BL/6 stimulators (*P* = 0.919). However, the mice with *in utero* saline injection (IU NS, *n* = 3) were both responsive to C57BL/6 (*P* = 0.004) and BALB/C alloantigens (*P* = 0.005). Lymphocytic proliferative responses to donor-specific C57BL/6 stimulators also reached significant difference between IU B-cells and IU NS (*P* = 0.005). Nil: no stimulator added.

Within 24 h after the assessment of donor B-cell chimerism, FVB/N recipients were subjected to donor skin transplantation for the evaluation of donor graft tolerance. FVB/N recipients kept donor skins longer than saline controls, but eventually rejected all the donor skins within 2–4 weeks (Figure 6A).

### Mixed Lymphocyte Reaction of FVB/N Lymphocytes after *In Utero* Injection of C57BL/6 B-Cells

We further collected five FVB/N recipients with *in utero* injection of enriched C57BL/6 B-cells for evaluating their *in vitro* lymphocyte response to allogeneic C57BL/6 cells by mixed lymphocyte reaction. The recipients’ lymphocytes were unresponsive to donor-specific C57BL/6 alloantigens, but significantly reacted with third-party BALB/C alloantigens (Figure 6B).

## Discussion

In this study, we used two inocula, soluble MHC exosomes and B-cells (cell surface MHC), to reappraise whether early *in utero* contact with alloantigens could cause allo-tolerance. Exosomes from APCs contain large amount of MHC class I/II molecules (17, 20, 26) and are immunologically active (27–30). Their presentation before transplantation has been shown to either modulate graft rejection (31) or improve allograft survivals and even facilitate induction of donor-specific tolerance (32). It provides the basis for the hypothesis that MHC exosomes may be a good substitute for allogeneic cells to prenatally induce allo-tolerance. *In utero* injection of exosomes was carried out in gestational day 14 murine fetuses. Considering that murine T-cell receptors were first expressed around gestational day 17 (33, 34), these murine fetal recipients fell well into a pre-immune category unable to mount adaptive immunity. Therefore, our approach had no misjudgment of an appropriate tolerization window for antigen exposure (35, 36). Our study showed that *in utero* exposure to allogeneic MHC exosomes could eliminated the donor-specific proliferative response of recipients’ T-cells but failed to confer donor skin tolerance. Although the exact mechanism by which exosomes modulate T-cell responses in this study remained unknown, it might be due to clonal deletion of alloreactive T-cells following their *in utero* exposure (37, 38). In addition, donor-derived exosomes might lessen anti-donor T-cell responses (31), presumably due to the generation of donor antigen-specific regulatory T-cells (39, 40).

In sharp contrast to MHC exosomes, marrow inocula had been demonstrated to induce allo-tolerance following their *in utero* injection (6, 41, 42). It raised the question of whether BMCs compared favorably in prenatal tolerance induction with soluble MHC exosomes. As we knew, marrow inocula contained hematopoietic stem cells whose engraftment in fetal recipients might yield significant levels of hematopoietic chimerism (6, 42, 43). Notably, the presence of hematopoietic chimerism itself has long since been linked to allo-tolerance after marrow or organ transplantation (44, 45). This made it difficult to tell whether prenatal tolerance induction by BMCs resulted from an event of *in utero* contact with allogeneic marrows or the establishment of hematopoietic chimerism. Thus, the use of marrow inocula certainly complicated the interpretation or analyses for underlying mechanisms that caused tolerance. To investigate prenatal allo-tolerance induction without the interference from hematopoietic stem cell engraftment, we had ever subjected murine fetuses to the injection of splenic lymphocytes (11). However, a great number of recipients succumbed to graft-versus-host disease before we could evaluate skin tolerance. As for the remaining recipients, allogeneic lymphocytes had no substantial capacity for conferring significant hematopoietic chimerism and skin graft tolerance. In this study, highly enriched B-cells were employed as *in utero* inocula to avoid graft-versus-host effects of allogeneic T-cells. This strategy could achieve low-level B-cell chimerism. However, it still failed to induce full tolerance to donor skin grafts despite that B-cell doses were comparable to BMC doses used in our previous studies (6, 42). Notably, B-cell inocula could only extend the survivals of skin allografts by a few days even though recipients’ lymphocytes were unresponsive *in vitro* specifically to donor alloantigens.

It is well known that allograft rejection results from a complex process of immune interactions involving the coordination of innate and adaptive immune responses. There are a wide variety of transplantation antigens responsible for allograft rejection, including MHC molecules, minor histocompatibility antigens, and ABO blood group antigens. Surface MHC molecules on donor cells are considered as the key target of transplantation immune responses, whereas minor histocompatibility antigen can be any donor non-MHC proteins that are processed, fractionated into peptides, and then bound to recipient APCs’ MHC molecules to elicit anti-graft T-cell responses. Therefore, the nature and magnitude of T-cell responses induced by alloantigen recognition in association with factors intrinsic to the grafts might affect the outcome of transplantation (46). In this study, both exosomal and B-cell alloantigens abolished donor-specific alloreactivity of recipients’ T-cells. However, neither could pass the most stringent tolerance test of skin grafting. This phenomenon might be attributed to minor histocompatibility antigens that were not included in exosomes or B-cells, but rather expressed on transplanted skins to trigger skin rejection.

The concept of actively acquired tolerance has fascinated immunological communities for more than half a century and attracted a number of laboratory work to replicate this immunological phenomenon (47). A review of studies for fetal/neonatal tolerance induction by allogeneic cells or simple peptides in the 1950s and 1960s revealed that these experiments might not always be conducted or analyzed in a sophisticated way. For example, the strain combination for allo-tolerance induction displayed few or even absent MHC barriers (1, 4, 5). Tolerance to soluble peptide antigens was determined simply by either delayed clearance of antigens injected (48), or decreased percentage of fatal anaphylaxis to a challenge with neglecting the underlying mechanism behind the shock in individual mice (49). To find fault with these studies might not be justified because they were the best the researchers could do at that time with insufficient immunological knowledge pertaining to graft rejection, and limited laboratory tools to explore an immunological phenomenon. In the 1990s with a clear understanding of MHC’s central role in transplantation rejection (2, 50) and the T-cell ontogeny (33, 34), *in utero* tolerance induction was reassessed using fully MHC-disparate BMCs in murine fetal recipients (41). It showed that donor-specific skin tolerance did not universally develop, but only succeeded in a minority of fetal recipients that obtained a certain level of hematopoietic chimerism. From that time onward, there was no shortage of work that failed to induce allo-tolerance or on the contrary, came up with conflicting evidence of immunization in the fetal (35, 51–53) as well as neonatal recipients (54–56). Although these scattered examples of inconsistent or even opposite results have clouded the picture of fetal or neonatal tolerance induction (35, 36), Medawar’s concept continued to reign as an unwavering immunological paradigm in terms of tolerance induction through antigen exposure before T-cell maturation.

Having worked on fetal tolerization over the years, we came to question aspects of “actively acquired tolerance” theory especially when our attempt at allergen desensitization through *in utero* ovalbumin exposure ended up with an event of *in utero* sensitization (57). Such an unexpected result could be attributed to macrophage-like fetal phagocytes that sequestered endocytosed ovalbumin for delayed antigen presentation. It was an important step forward toward the understanding of how fetal immune system was shaped following antigen exposure before its full development. Apparently, innate fetal phagocytes played a critical role in dealing with antigens at the very beginning when the antigens were introduced to the fetuses to initiate an event of *in utero* contact. They had an important implication for fetal immunological consequences in responses to antigen exposure regardless of T-cell immaturity.

There is no doubt that the intricacy of alloantigen recognition by the immune system (46, 50) is far beyond what we have known about the immune recognition of soluble ovalbumin antigen. *In utero* exposure to alloantigens from MHC exosomes or B-cells in this study or BMCs in our previous studies (6, 42) could abolish alloreactivity of recipients’ lymphocytes, but might not always render fetal recipients tolerant to donor skins. Although minor antigens expressed by donor skins instead of exosomal/B-cell inocula might lead to donor skin rejection in the presence of T-cell unresponsiveness, a wide range of donor skin survivals following *in utero* BMC injection was not explicable in terms of minor antigens (6, 42). The same batch of allogeneic BMCs might endow fetal recipients of the same litter with variable results, ranging from no observed to complete chimerism. Notably, donor skin tolerance was found to closely relate to hematopoietic chimerism generated rather than exposure intensity (doses) of donor BMCs given to the fetal recipients (6). Namely, skin tolerance was not an event of “all or none,” but rather a graded phenomenon predicted by hematopoietic chimerism. More specifically, complete skin tolerance was only conditional on peripheral chimerism meeting a threshold level at skin graft placement (42). Such a scenario was also reported by many researchers (37, 38, 41). As a result, we found it difficult to disregard the importance of hematopoietic chimerism for skin tolerance. Using MHC exosomes and B-cells in this study, we were able to investigate the immunological outcomes of *in utero* exposure to alloantigens independently of the influence of hematopoietic stem cell engraftment. It disclosed that donor skin survivals never reached a level of enduring tolerance. However, it is noteworthy that B-cell inocula could generate microchimerism and lead to delayed skin rejection, similar to BMC inocula (6). Taken together, these findings might dawn on researchers in the field that hematopoietic chimerism had an indispensable role in facilitating skin graft survivals. Thus, it is difficult to reconcile these observations with the postulated mechanism of a simple early *in utero* contact, whereby a pre-immune fetus achieved “actively acquired tolerance.”

*In utero* exposure to alloantigens could abolish alloreactivity of recipients’ lymphocytes, but not always induce donor skin tolerance. Hematopoietic chimerism, if generated by cell inocula, might facilitate donor skin survivals, ranging from prolonged for a few days by B-cells in this study to persistent for ≥4 months by BMCs (6, 42). Thus, BMCs containing hematopoietic stem cells might represent a unique kind of *in utero* inocula, capable of conferring significant hematopoietic chimerism and long-lasting skin tolerance. Taken together with the discovery of *in utero* sensitization to soluble peptides of ovalbumin (57), the classical school of thought claiming that an early enough *in utero* contact with foreign antigens caused tolerance might oversimplify the situation. Conclusively, the immunological outcome of fetal exposure to foreign antigens might vary according to the type or nature of antigens introduced.

## Ethics Statement

Mice were housed in the Animal Care Facility at Chang Gung Memorial Hospital (CGMH) under the standard guidelines from “Guide for the Care and Use of Laboratory Animals” and with the approval of the CGMH Committee on Animal Research.

## Author Contributions

J-CC performed *in utero* injection, analyzed the data, prepared the figures, and wrote the manuscript. L-SO performed cell culture, fractionated exosomes, and measured lymphocyte proliferation. C-CC performed animal surgery. M-LK helped with data analyses and manuscript preparation. L-YT and H-LC assisted in experiments and animal surgery and care.

## Conflict of Interest Statement

The authors declare that the research was conducted in the absence of any commercial or financial relationships that could be construed as a potential conflict of interest.
